# Generation and Imaging of Transgenic Mice that Express G-CaMP7 under a Tetracycline Response Element

**DOI:** 10.1371/journal.pone.0125354

**Published:** 2015-05-06

**Authors:** Masaaki Sato, Masako Kawano, Masamichi Ohkura, Keiko Gengyo-Ando, Junichi Nakai, Yasunori Hayashi

**Affiliations:** 1 RIKEN Brain Science Institute, Wako, Saitama, Japan; 2 PRESTO, Japan Science and Technology Agency, Kawaguchi, Saitama, Japan; 3 Brain Science Institute, Saitama University, Saitama, Japan; Tokyo University of Agriculture, JAPAN

## Abstract

The spatiotemporally controlled expression of G-CaMP fluorescent calcium indicator proteins can facilitate reliable imaging of brain circuit activity. Here, we generated a transgenic mouse line that expresses G-CaMP7 under a tetracycline response element. When crossed with a forebrain-specific tetracycline-controlled transactivator driver line, the mice expressed G-CaMP7 in defined cell populations in a tetracycline-controlled manner, notably in pyramidal neurons in layer 2/3 of the cortex and in the CA1 area of the hippocampus; this expression allowed for imaging of the *in vivo* activity of these circuits. This mouse line thus provides a useful genetic tool for controlled G-CaMP expression *in vivo*.

## Introduction

Genetically encoded calcium indicators (GECIs), such as green fluorescent protein (GFP)-based G-CaMPs, are increasingly used to monitor neural activity in living brains [1–6]. GECIs have recently begun to replace synthetic calcium indicator dyes because GECIs can be expressed in genetically defined cell populations to perform long-term repeated imaging of activity in the same cell populations [7–10]. GECIs can be introduced into cells by gene transfer techniques, such as *in utero* electroporation, viral vectors and transgenic technologies. Once good lines are established, transgenic mice have significant advantages, such as the capability for GECI labeling of each animal without surgery and for highly reproducible and homogeneous GECI expression; these advantages facilitate a more reliable and efficient collection of imaging data on the activity of large cell populations.

To date, several transgenic mouse lines that express G-CaMPs in the brain have been reported [11–15]. The different G-CaMP variants expressed in these mice have different sensitivities, kinetics and signal-to-noise ratios, which allows researchers to monitor a wide range of neural activity [2, 3, 5]. Although cell-type-specific expression can be achieved via Cre/lox-mediated recombination, the temporal control of G-CaMP expression, which could circumvent the potential adverse effects of long-term and/or developmental expression of G-CaMPs, is currently limited to ligand-induced recombination [12, 16, 17]. The tetracycline-controlled transactivator (tTA)/tetracycline response element (TRE) system offers an attractive option to address this need and enables the utilization of currently available resources, the various tTA driver lines that target specific cell populations [18]. We thus generated and characterized transgenic mice that express G-CaMP7 under a TRE.

## Materials and Methods

### Ethics statement

This study was carried out in accordance with the institutional guidelines and protocols approved by the RIKEN Animal Experiments Committee and Genetic Recombinant Experiment Safety Committee (approval numbers: H25-2-207(5) and 2012-015(22)).

### Generation of TRE-G-CaMP7 mice

The cDNA encoding G-CaMP7 [4] connected to the coding sequence of DsRed2 via the 2A peptide sequence from the *Thosea asigna* virus (T2A) was subcloned between the BamHI and NotI sites of a modified pTRE-Tight vector (Clontech). The 3.5 kb DNA fragment excised by the ApaI and SphI restriction enzymes was gel-purified and injected into the pronuclei of fertilized eggs of C57BL/6J mice using standard techniques. The mice were genotyped using PCR to amplify DNA samples extracted from their tails. The primers used were 5’-CTGCTGCCCGACAACCA-3’ and 5’-GTCGTCCTTGAAGAAGATGG-3’, which amplified a 465-bp fragment of the G-CaMP7 coding sequence from the DNA samples of transgene-positive mice. The mouse line, termed TRE-G-CaMP7, is available at the RIKEN BioResource Center (http://www.brc.riken.jp/lab/animal/en/; stock number RBRC06510).

### Analysis of transgene expression

Double transgenic mice obtained by breeding TRE-G-CaMP7 mice with driver mice expressing tTA under the forebrain-specific calcium/calmodulin-dependent protein kinase IIα promoter (CaMKII-tTA mice; stock number 003010, Jackson Laboratory) [19] were deeply anesthetized with Avertin and were perfused transcardially with phosphate-buffered saline (PBS) followed by 4% paraformaldehyde (PFA) in PBS. Brains were removed and further fixed in 4% PFA at 4°C overnight. Coronal or parasagittal sections were cut on a vibratome to a thickness of 50 or 100 μm, respectively. For immunolabeling, the sections were incubated at 4°C overnight with mouse anti-calcium/calmodulin-dependent protein kinase IIα (CaMKIIα) antibody (1:1000, Clone 6G9, Millipore), rabbit anti-Iba1 antibody (1:2000, 019–19741, Wako Pure Chemical Industries, Ltd., Japan), rabbit anti-glial fibrillary acidic protein (GFAP) antibody (1:1000, N1506, Dako) or anti-c-fos antibody (1:5000, PC38, Calbiochem) diluted in PBS containing 2% normal goat serum, 1% BSA and 0.1% Triton X-100, followed by Alexa Fluor 647-labeled goat anti-rabbit or anti-mouse IgG antibody (1:700–1:1000, A-21245 or A-21236, Life Technologies) diluted in the same buffer at room temperature for 1 h. Nuclear counterstaining was performed in PBS containing 10 μg/ml Hoechst 33258 (Calbiochem) and 0.1% Triton X-100 at room temperature for 5 min. Fluorescence images were acquired using a Keyence BZ-9000 epifluorescence microscope equipped with a 4x objective or an Olympus FV1000 or FV1200 laser-scanning confocal microscope equipped with a 4x, 10x, 20x dry or 60x oil immersion objective. The G-CaMP7 and DsRed2 signals shown in all the images represent their native fluorescence and were not enhanced by immunolabeling. For cell counting, the fraction of G-CaMP7-labeled cells was calculated against the number of total neuronal cells stained with NeuroTrace 435/455 Blue Fluorescent Nissl Stain (1:100, Life Technologies) diluted in PBS containing 0.1% Triton X-100 at room temperature for 20 min. G-CaMP7-labeled cells and NeuroTrace-stained cells were counted in upper layer 2/3 (< 250 μm from the pia), lower layer 2/3 (> 250 μm from the pia) and layer 5 of the neocortex as well as the CA1 area of the hippocampus. More than 200 cells in 6–8 fields per animal (field size 212 x 212 μm for the hippocampus and 318 x 318 μm for the cortex; 16–58 cells per field) were counted for each layer or area. For doxycycline (Dox) treatment, mice were housed with *ad libitum* access to 0.1% saccharin water with or without 2 mg/ml doxycycline hyclate (Sigma-Aldrich) for 2–4 weeks. For withdrawal from Dox, the mice treated with Dox for 2 weeks were then given water without Dox for an additional 2 weeks. Light-induced c-fos expression in the visual cortex was examined in mice kept in darkness for 24 h and then exposed to ambient light for 1 h before perfusion. Control mice were perfused immediately after 24 h of adaptation to darkness.

### *In vivo* two-photon imaging and data analysis

Adult double-transgenic mice were anesthetized with isoflurane (3% induction, 1.5% maintenance) and were placed in a stereotaxic frame. Atropine (0.3 mg/kg, s.c.) and dexamethasone (2 mg/kg, s.c.) were administered prior to the anesthesia to reduce respiratory secretions and brain edema, respectively [20]. A stainless steel head plate with a circular opening (7 mm diameter) was placed over the left parietal bone and attached to the skull with dental acrylic. Craniotomy and window preparation for cortical and hippocampal imaging were performed essentially as described by Holtmaat et al. and Dombeck et al., respectively [7, 21]. For hippocampal imaging, a small volume of cortical tissue overlying the dorsal CA1 region of the hippocampus was surgically removed by aspiration before the implantation of an imaging window [7]. After surgery, the mice were returned to their home cages and were allowed to recover until the imaging experiments.

On the day of imaging, each mouse was re-anesthetized with isoflurane (3% induction, 1% maintenance) supplemented with chlorprothixene (1 mg/kg, i.p.) and was placed under the microscope objective via the head plate. Body temperature was maintained at 37°C with a heating pad throughout the imaging sessions. G-CaMP7 was excited using a Ti:Sapphire laser (Mai Tai DeepSee eHP, Spectra-Physics) at 910 nm, and time-series fluorescence changes in neurons during spontaneous network activity in the dorsal posterior cortex (visual cortex) [20] or the dorsal CA1 hippocampus [7] were imaged using a 495–540 nm bandpass filter and a GaAsP photomultiplier tube. The laser power under the objective was 10–28 mW. Images were acquired using an Olympus FV1000MPE microscope equipped with a 25x NA 1.05 objective (Olympus) in 256 x 256 pixels (field size 254 x 254 μm for the cortex and 169 x 169 μm for the hippocampus) or 128 x 128 pixels (12.6 x 12.6 μm for dendritic imaging) at a frame rate of 2.3 or 5.3 Hz (pixel dwell time 2 μs/pixel), respectively. For dual color imaging, DsRed2 was simultaneously excited at 910 nm, and the signal was separated by a 570 nm dichroic mirror and a 575–630 nm bandpass filter.

We performed image analysis using custom software written in MATLAB (MathWorks). The center of a region of interest (ROI) in each cell was determined manually on the time-averaged G-CaMP7 fluorescence image so that the ROI was defined as a circle with a 6-pixel (6.0 μm for cortical neurons) or 10-pixel (6.6 μm for hippocampal neurons) radius inscribed within the cell body. The fluorescence intensity values of the pixels within the ROI in each frame of the time-lapse image sequences were then averaged to represent the cellular signal *F* for that cell. For each *F*, a baseline value (*F0*) was defined as the mean of the 75th (for cortical imaging) or 80th percentile (for hippocampal imaging) of all the data points. *F0* was then used to calculate Δ*F/F* as (*F-F0)/F0*. The timing and magnitude of neuronal activity were defined as the local maxima of Δ*F/F* above the threshold (4 standard deviations from *F0*). The center of an ROI for the cortical neuropil was also defined manually in image areas that were visually devoid of cell bodies; this ROI had the same size and shape as the ROI used in the analysis of cellular signals. An ROI for a basal dendritic segment of CA1 pyramidal cells was defined manually. A pair-wise cross-correlation analysis of Δ*F/F* signals was performed using custom software written in MATLAB. For hippocampal imaging, the sequential activation of a pair of cells was quantified by counting the number of events in which one cell’s activity preceded or followed another’s by one image frame. Quasi-ratiometric measurement was performed by calculating the normalized ratio of G-CaMP7 to DsRed2 signals. To simulate motion artifacts, randomly generated image displacements of 8–12 pixels (0.79–1.18 μm) in distance and 0.19–0.56 s in duration were added to the real data at an average frequency of 0.2 Hz. The direction of the artificial motion artifacts was chosen randomly and remained fixed throughout the simulation.

## Results and Discussion

**Fig 1:**
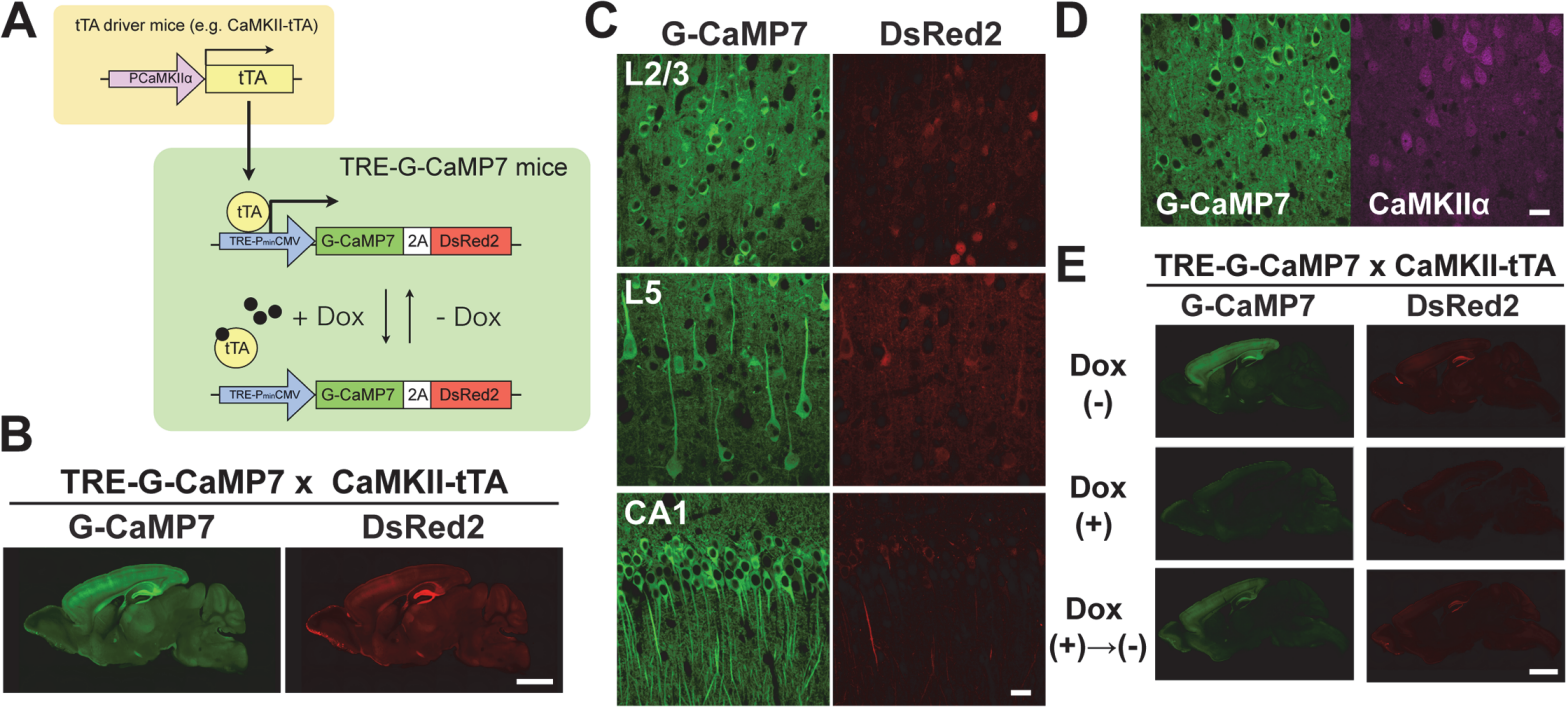
Transgene expression in TRE-G-CaMP7 mice crossed with CaMKII-tTA driver mice. **A**, Design and strategy for controlled G-CaMP7 expression in TRE-G-CaMP7 mice. G-CaMP7 expression is controlled spatially and temporally by a cell-type-specific tTA driver mouse line and doxycycline (Dox) administration, respectively. PCaMKIIα, CaMKIIα promoter; PminCMV, minimal cytomegalovirus promoter. **B**, Expression patterns of G-CaMP7 and DsRed2 in 1-month-old mice. Scale bar = 2 mm. **C**, Expression of G-CaMP7 and DsRed2 in layer 2/3 (L2/3) and layer 5 (L5) pyramidal neurons in the visual cortex and in CA1 pyramidal neurons in the hippocampus. Scale bar = 20 μm. **D**, Expression of G-CaMP7 in CaMKIIα-positive pyramidal neurons in layer 2/3 of the visual cortex. Scale bar = 20 μm. **E**, Reversible control of transgene expression by Dox administration. Parasagittal sections were cut from mice without Dox administration (Dox (-)), after 2 weeks of Dox administration (Dox (+)), and after 2 weeks of Dox withdrawal (Dox (+)→(-)). All the sections were cut from mice at 4–5 months of age, and the images were obtained with the same exposure time. Scale bar = 2 mm.

We generated a transgenic mouse line that expresses G-CaMP7 and DsRed2 via 2A peptide-mediated bicistronic expression under the control of a TRE (Fig 1A). G-CaMP7 is a recently improved G-CaMP variant that exhibits large fluorescence changes in response to a broad range of intracellular calcium concentrations and is thus suitable for reliable *in vivo* calcium imaging [4]. The coexpressed DsRed2 serves as a calcium-independent fluorescent marker protein of a different color and aids the identification of G-CaMP7-expressing cells *in vivo* because the basal fluorescence of G-CaMP7 is relatively low [4]. In addition, this DsRed2 signal can be used as a normalization factor for the quasi-ratiometric measurement of calcium responses and to estimate the extent of image motion artifacts, especially when imaging is performed under awake behaving conditions (for an example of ratiometric measurements, see S5 Fig). From 774 DNA-injected eggs, 12 transgene-positive founder lines were obtained, 7 of which showed detectable levels of G-CaMP7 fluorescence in brain sections of offspring crossed with the CaMKII-tTA line. By contrast, our parallel effort to generate CaMKII-G-CaMP7 transgenic mice by inserting the same bicistronic expression cassette under the CaMKIIα-promoter [19] produced 7 transgene-positive mice from 1151 DNA-injected eggs. None of these mice showed detectable G-CaMP7 fluorescence, suggesting that the tTA/TRE system may have achieved higher expression levels by transcriptional amplification.

We more thoroughly characterized one founder line that expressed G-CaMP7 and DsRed2 prominently in the forebrain by crossing the founder line with the CaMKII-tTA line (TRE-G-CaMP7 x CaMKII-tTA mice; Fig 1B). Higher-magnification images revealed that pyramidal neurons in layer 2/3 and layer 5 of the neocortex as well as pyramidal neurons in the CA1 area of the hippocampus strongly expressed G-CaMP7 with varying degrees of DsRed2 coexpression (Fig 1C). The coexpressed DsRed2 exhibited cytoplasmic localization in most cells, although it was also localized to the nucleus in a subset of cells, particularly in layer 2/3 of the cortex (Fig 1C). The fractions of G-CaMP7-expressing cells among the total neurons were 49.4±4.7, 62.5±6.5, 45.1±6.9 and 71.5±5.2% in upper layer 2/3, lower layer 2/3, and layer 5 in the cortex and the CA1 area of the hippocampus, respectively (mean±SD, n = 13–15 fields, 2 mice). Immunofluorescence labeling confirmed that G-CaMP7-expressing neurons in these mice were CaMKIIα-positive, consistent with the expression pattern of tTA defined by the cell-type-specific promoter of the driver line (Fig 1D) [19].

In the hippocampus, a subset of CA3 pyramidal cells and dentate gyrus granule cells appeared to be less strongly labeled with G-CaMP7 than the CA1 pyramidal cells, whereas subicular pyramidal neurons were more strongly labeled (S1A Fig). Strikingly, the stratum lacunosum-moleculare and the molecular layer of the dentate gyrus were intensely labeled with G-CaMP7 and DsRed2 (S1A Fig). This finding was in accordance with the strong labeling of the neurons in the entorhinal cortex, which projects perforant path fibers to these layers (S1B Fig).

Previous studies have reported that G-CaMPs expressed by adeno-associated viral vectors accumulated within the cytoplasm and the nucleus over time; these cells exhibited abnormal calcium responses [2, 7]. The G-CaMP7-expressing cells in the TRE-G-CaMP7 x CaMKII-tTA mice showed no apparent abnormal morphology, and their nuclei were devoid of G-CaMP7 fluorescence even at 7 months of age (S2 Fig).

The functional influence of long-term G-CaMP7 expression was examined histologically by visualizing the light-induced expression of the immediate early gene product c-fos in the visual cortices of TRE-G-CaMP7 x CaMKII-tTA and wild-type mice at 8 months of age (S3 Fig). Exposing mice of both genotypes to light for 1 h caused robust induction of c-fos protein expression in the nuclei of many neurons in the visual cortex. The levels of induction were equivalent in both genotypes, suggesting that neuronal function remained undisturbed after long-term transgenic G-CaMP7 expression in these mice.

The possibility of neuronal toxicity associated with long-term transgenic labeling with G-CaMP7 was further assessed by immunofluorescence microscopy using antibodies against the reactive astrocyte marker GFAP and the microglial marker Iba1 (S4 Fig). Most of the GFAP-positive astrocytes were located in the hippocampus, but only a small number of them were observed in the neocortex in both TRE-G-CaMP7 x CaMKII-tTA and wild-type mice at 7 months of age (S4A Fig), whereas Iba1-positive microglia were diffusely distributed throughout the neocortex and hippocampus (S4B Fig). The finding that the distribution patterns of GFAP-positive astrocytes and Iba1-positive microglia were indistinguishable between both genotypes of mice provides evidence that long-term transgene expression is non-toxic in older TRE-G-CaMP7 x CaMKII-tTA mice. Moreover, the absence of G-CaMP7 expression in these two types of glial cells further confirms the neuronal expression of G-CaMP7 in the TRE-G-CaMP7 x CaMKII-tTA mice (S4C Fig).

We next tested whether G-CaMP7 expression could be reversibly suppressed by the administration of the tetracycline derivative doxycycline (Dox; Fig 1A). G-CaMP7 and DsRed2 fluorescence signals in the forebrain were reduced nearly to the background level in TRE-G-CaMP7 x CaMKII-tTA mice treated with Dox (2 mg/ml in 0.1% saccharin water) for 2–4 weeks (Fig 1E). Withdrawal from Dox for 2 weeks after 2 weeks of treatment restored the G-CaMP7 and DsRed2 signals to the control level (Fig 1E), demonstrating that temporal control of G-CaMP7 expression is possible in these mice.

**Fig 2:**
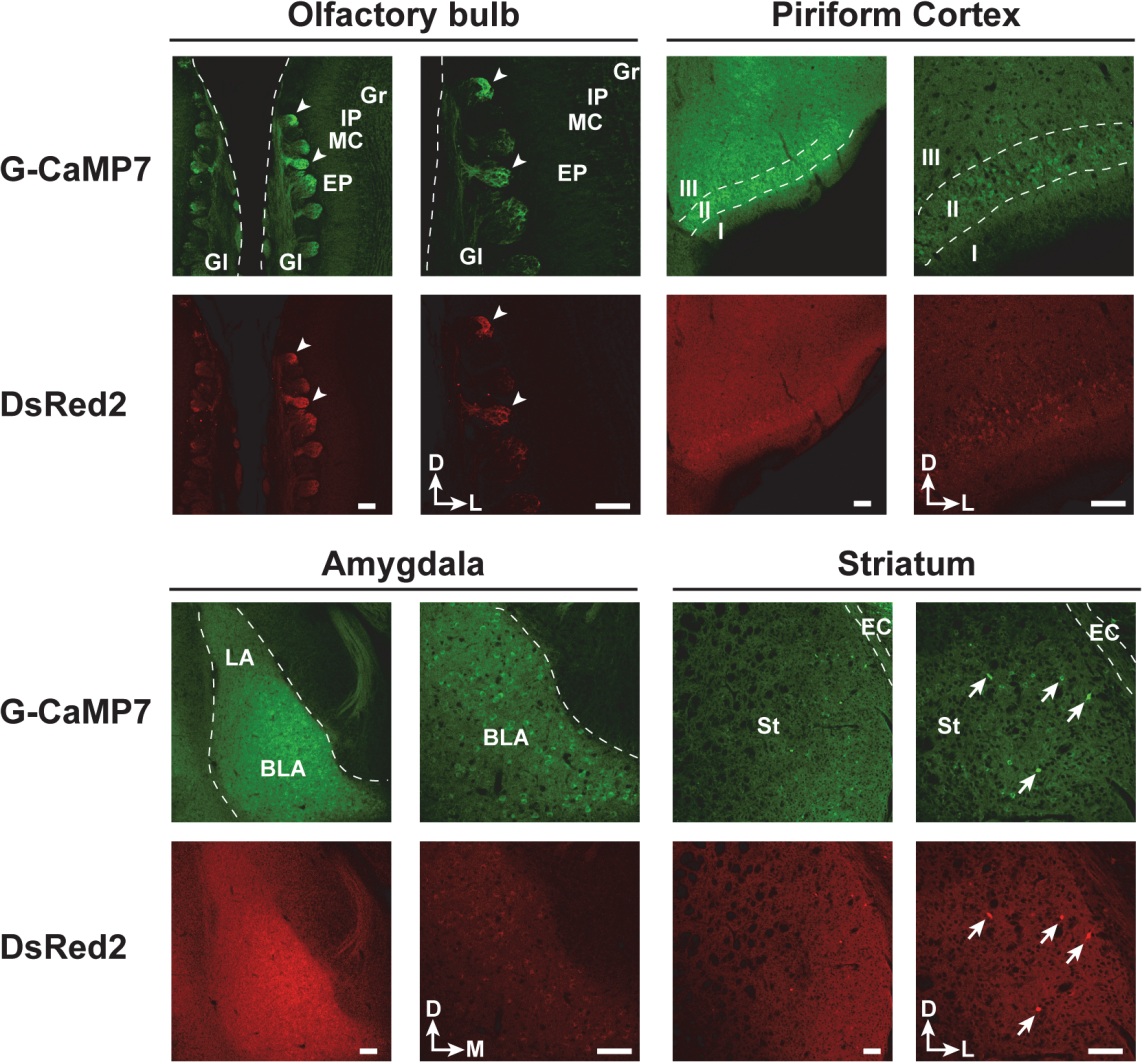
Transgene expression in brain areas outside the neocortex and hippocampus. The expression patterns of G-CaMP7 and DsRed2 in coronal sections of the olfactory bulb, piriform cortex, amygdala and striatum from TRE-G-CaMP7 x CaMKII-tTA mice are shown at lower (left panels for each brain area) and higher (right panels) magnifications. Arrowheads indicate olfactory glomeruli intensely labeled by G-CaMP7. Arrows represent examples of striatal neurons strongly expressing G-CaMP7. Gl, glomerular layer; EP, external plexiform layer; MC, mitral cell layer; IP, internal plexiform layer; Gr, granule cell layer; I, II, and III, layers I, II, and III of the piriform cortex, respectively; LA, lateral amygdala; BLA, basolateral amygdala; St, striatum; EC, external capsule; D, dorsal; L, lateral; M, medial. Scale bar = 100 μm.

In addition to the neocortex and hippocampus, TRE-G-CaMP7 x CaMKII-tTA mice exhibited notable G-CaMP7 expression in various neuronal populations in forebrain structures, such as the axon terminals of olfactory receptor neurons in olfactory glomeruli, layer 2 neurons in the piriform cortex and neurons in the basolateral amygdala (Fig 2). A subset of neurons in the lateral part of the striatum was also found to be labeled with G-CaMP7 (Fig 2). These expression patterns further expand the applicability of these mice in *in vitro* and *in vivo* imaging studies of neural circuit activity in widespread forebrain areas.

**Fig 3:**
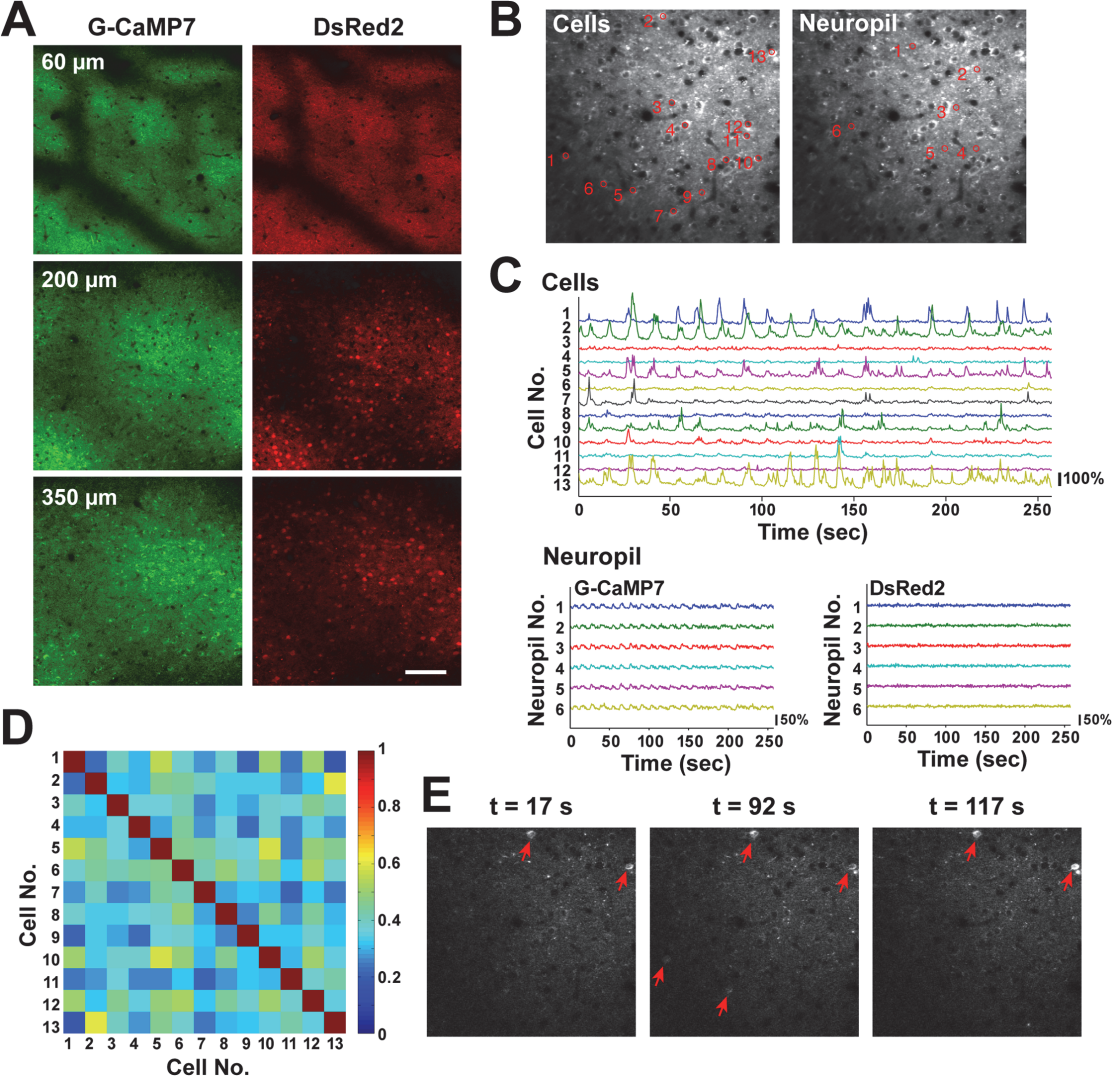
Two-photon imaging of spontaneous cortical layer 2/3 circuit activity in anesthetized TRE-G-CaMP7 x CaMKII-tTA mice at 4 months of age. **A**, Neurons expressing G-CaMP7 (left) and DsRed2 (right) were imaged in the posterior cortex 60, 200, and 350 μm from the pial surface. The darker areas in each image are shadows of vessels. Scale bar = 100 μm. **B**, The positions and numbers of 13 active neurons are indicated in an average G-CaMP7 fluorescence image acquired at a depth of 200 μm (left). Similarly, those of 6 neuropil areas are shown in the same average G-CaMP7 fluorescence image (right). **C**, Baseline-normalized G-CaMP7 fluorescence time traces for the same 13 cells (top) and 6 neuropil regions (bottom left) and baseline-normalized DsRed2 fluorescence time traces for the same 6 neuropil regions (bottom right). **D**, A cross-correlation matrix of G-CaMP7 fluorescence time traces of the 13 cells. **E**, Example time-lapse images of G-CaMP7 fluorescence during spontaneous network activity in layer 2/3 of the cortex. Cell 2 and Cell 13 (as designated in **B**) were synchronously activated multiple times. Time stamps are indicated above the images. Active cells are indicated by red arrows.

**Fig 4:**
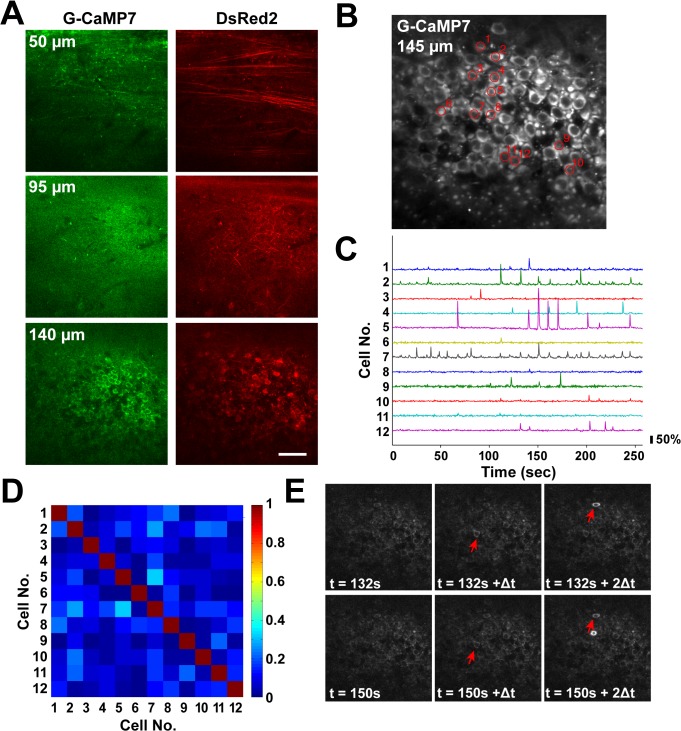
Two-photon imaging of spontaneous hippocampal CA1 circuit activity in anesthetized TRE-G-CaMP7 x CaMKII-tTA mice at 4 months of age. **A**, Different subcellular compartments of pyramidal neurons expressing G-CaMP7 (left) and DsRed2 (right) were imaged in the dorsal CA1 hippocampus 50, 95 and 140 μm from the hippocampal surface. Scale bar = 50 μm. **B**, The positions and numbers of 12 active neurons selected for analysis are indicated in an average G-CaMP7 fluorescence image acquired at a depth of 145 μm. **C**, Baseline-normalized G-CaMP7 fluorescence time traces of the 12 active cells. **D**, A cross-correlation matrix of G-CaMP7 fluorescence time traces of the 12 active cells. **E**, Example time-lapse images of G-CaMP7 fluorescence during spontaneous network activity in the CA1 hippocampus. Cells 7 and 2 (as designated in **B**) were sequentially activated multiple times. Only two out of nine total sequential activation events are shown here. Time stamps are indicated at the bottom of the images. Δt represents the image sampling interval (0.43 s). Active cells 7 and 2 are indicated by red arrows.

The functionality and utility of the TRE-G-CaMP7 x CaMKII-tTA mice were further demonstrated using two-photon imaging of spontaneous circuit activity in the neocortex and hippocampus of anesthetized mice (Figs 3 and 4). In the neocortex, a large population of neurons with homogeneous G-CaMP7 and DsRed2 labeling were imaged in superficial layers of different depths (Fig 3A and S1 Movie). Time-lapse imaging revealed that a subpopulation of layer 2/3 neurons exhibited large, spontaneous calcium transients, which often occurred near the peaks of synchronous slow baseline oscillations (Fig 3B and 3C and S2 Movie). These correlated slow calcium oscillations were also observed in G-CaMP7 but not in DsRed2 signals in the neuropil and were similar to those observed in a cortex loaded with the synthetic calcium dye Oregon Green BAPTA-1 AM [22] (Fig 3C). A pair-wise correlation analysis of cellular G-CaMP7 signals resulted in an average correlation coefficient of 0.308±0.143 (Fig 3D; mean±SD, n = 129 cell pairs, 3 mice), which was indicative of a weak overall correlation, and the identification of a small number of cell pairs that were anatomically remote but exhibited highly correlated spontaneous activity (Fig 3E).

In the CA1 area of the hippocampus, different subcellular compartments, such as axons, basal dendrites and cell bodies, of G-CaMP7- and DsRed2-labeled pyramidal cells were imaged at different depths corresponding to the alveus, stratum oriens and stratum pyramidale (Fig 4A and S3 Movie). Time-lapse imaging revealed that a subset of pyramidal cells exhibited large, spontaneous calcium transients with no baseline oscillations (Fig 4B and 4C and S4 Movie). The average pair-wise correlation coefficient was 0.030±0.066 (mean +/- SD, n = 355 cell pairs, 3 mice), suggesting that spontaneous network activity was much less correlated in the hippocampus than in the cortex (Fig 4D). However, the sequential activation of particular cell pairs was repeatedly observed in the hippocampus (Fig 4E), suggesting the presence of hidden temporal structures [23–25]. Calcium transients could be imaged not only in cell bodies but also in the basal dendrites of the CA1 pyramidal cells in these mice (S5 Fig) [26]. Collectively, the results shown in Figs 3 and 4 demonstrate that TRE-G-CaMP7 x CaMKII-tTA mice can be used to study the activity patterns of neuronal ensembles *in vivo*. A full elucidation of the patterns observed in this work will require future in-depth studies.

In conclusion, we generated and characterized TRE-G-CaMP7 transgenic mice expressing a recently improved G-CaMP7 under a TRE. The expression of G-CaMP7 in these mice is homogeneous, stable and functional, and it can be controlled spatially and temporally by cell-type-specific tTA driver lines and Dox treatment, respectively. Together with a recently reported transgenic mouse line that expresses a Förster resonance energy transfer (FRET)-based GECI via a tTA-mediated strategy [27], our TRE-G-CaMP7 mice offer a useful alternative to Cre/lox-mediated cell-type-specific GECI labeling and will provide a valuable genetic tool for the reliable imaging of neural circuit activity *in vivo*.

## Supporting Information

S1 Fig Transgene expression in the hippocampus and medial entorhinal cortex of TRE-G-CaMP7 x CaMKII-tTA mice at 1 month of age. **A,** Expression patterns of G-CaMP7 and DsRed2 in a parasagittal section of the hippocampus. CA1, CA1 area of the hippocampus; CA3, CA3 area of the hippocampus; DG, dentate gyrus; ML, molecular layer of the dentate gyrus; SLM, stratum lacunosum-moleculare; S, subiculum. Scale bar = 500 μm. **B,** Expression patterns of G-CaMP7 and DsRed2 in a parasagittal section of the medial entorhinal cortex. Lower panels show higher-magnification images. HP, hippocampus; mEC, medial entorhinal cortex; I, II, and III, layers I, II, and III of the medial entorhinal cortex, respectively; S, subiculum; VC, visual cortex. Scale bar = 250 μm.
(TIF)

S2 Fig Transgene expression in TRE-G-CaMP7 x CaMKII-tTA mice at 7 months of age. **A,** Expression patterns of G-CaMP7 and DsRed2 in 7-month-old mice. Scale bar = 2 mm. **B,** G-CaMP7 expression and nuclear staining (Hoechst) of layer 2/3 (L2/3) pyramidal neurons in the visual cortex and CA1 pyramidal neurons in the hippocampus. Scale bar = 20 μm.
(TIF)

S3 Fig Light-induced c-fos expression in the visual cortex of TRE-G-CaMP7 x CaMKII-tTA and wild-type mice at 8 months of age. **A,** Light-induced c-fos expression in the visual cortex of TRE-G-CaMP7 x CaMKII-tTA mice at 8 months of age. The left, middle, and right panels show images of c-fos immunoreactivity, G-CaMP7 fluorescence and c-fos immunoreactivity overlaid with G-CaMP7 fluorescence, respectively. Top, images after 24 h of adaptation to darkness (Dark). Bottom, images after 24 h of adaptation to darkness followed by 1 h of exposure to light (Light). Scale bar = 200 μm. **B,** Light-induced c-fos expression in the visual cortex of wild-type mice (WT) at 8 months of age. Scale bar = 200 μm. **C,** Higher-magnification images of layer 2/3 visual cortical neurons expressing c-fos in response to light stimulation in TRE-G-CaMP7 x CaMKII-tTA mice at 8 months of age. Arrows indicate examples of cells that exhibited robust c-fos induction despite the presence of intracellular G-CaMP7 aggregates. Scale bar = 20 μm. **D,** Higher-magnification images of layer 2/3 visual cortical neurons expressing c-fos in response to light stimulation in wild-type mice at 8 months of age. Scale bar = 20 μm.
(TIF)

S4 Fig GFAP and Iba1 immunoreactivity in the neocortex and hippocampus of TRE-G-CaMP7 x CaMKII-tTA and wild-type mice at 7 months of age. **A,** Top, images of GFAP immunofluoresence (magenta) in the neocortex and hippocampus of TRE-G-CaMP7 x CaMKII-tTA and wild-type (WT) mice. Middle, GFAP immunofluorescence overlaid with Hoechst nuclear counterstaining (blue). Bottom, GFAP immunofluorescence overlaid with G-CaMP7 fluorescence (green) in TRE-G-CaMP7 x CaMKII-tTA mice. **B,** Top, images of Iba1 immunofluoresence (magenta) in the neocortex and hippocampus of TRE-G-CaMP7 x CaMKII-tTA and wild-type mice. Middle, Iba1 immunofluorescence overlaid with Hoechst nuclear counterstaining (blue). Bottom, Iba1 immunofluorescence overlaid with G-CaMP7 fluorescence (green) in TRE-G-CaMP7 x CaMKII-tTA mice. CA1, CA1 area of the hippocampus; CC, corpus callosum; Cx, neocortex; DG, dentate gyrus. Scale bar = 500 μm. **C**, Higher-magnification images of GFAP-positive astrocytes (left, arrowheads) and Iba1-positive microglia (right, arrowheads) in the CA1 area of the hippocampus overlaid with G-CaMP7 fluorescence. Scale bar = 20 μm.
(TIF)

S5 Fig Two-photon imaging of spontaneous basal dendritic activity in hippocampal CA1 pyramidal neurons in anesthetized TRE-G-CaMP7 x CaMKII-tTA mice. **A,** Basal dendrites of hippocampal CA1 pyramidal neurons labeled with G-CaMP7 and DsRed2 were imaged 80 μm from the hippocampal surface in a 6-month-old mouse. Magnified images of the areas enclosed by red dotted lines are shown to the right. The dendritic segment enclosed by the red line in the magnified G-CaMP7 image delineates the region of interest defined for the traces shown in **C**. Scale bar = 5 μm. **B,** Example time-lapse images of G-CaMP7 and DsRed2 fluorescence during spontaneous activity of a basal dendrite of a hippocampal CA1 pyramidal neuron. Δt represents the image sampling interval (0.19 s). **C,** Top, a trace of changes in G-CaMP7 fluorescence in the basal dendritic segment shown in **A** and **B**. The dendritic activity shown in **B** occurred at the time indicated by the arrow. Bottom, traces of G-CaMP7, DsRed2 and G-CaMP7/DsRed2 ratiometric signals of the same data, except for the addition of simulated motion artifacts. Artificial image displacements occurred at the random timings indicated by the inverted open triangles. Quasi-ratiometric calculations using DsRed2 signals effectively removed the baseline motion artifacts. Scale bar = 5 s (horizontal) and 20% change in the fluorescence intensity or the G-CaMP7/DsRed2 ratio (vertical).
(TIF)

S1 Movie Expression of G-CaMP7 and DsRed2 imaged in the neocortex of TRE-G-CaMP7 transgenic mice crossed with CaMKII-tTA driver mice. Images were acquired 0 to 350 μm from the pial surface at 10-μm intervals. Shown on the left and right are grayscale images of G-CaMP7 and DsRed2 signals, respectively. Depths are indicated in the upper left corner of the movie. The size of the imaged area is 509 x 509 μm (512 x 512 pixels).
(AVI)

S2 Movie Spontaneous activity of cortical circuits imaged using TRE-G-CaMP7 transgenic mice crossed with CaMKII-tTA driver mice. Images were acquired 200 μm below the surface of the posterior cortex. The first 300 frames (~129 s) of the data presented in Fig 3 are shown in this movie. The playback speed is 5x faster than the speed of real activity. Time stamps are indicated in the upper left corner of the movie. The size of the imaged area is 254 x 254 μm (256 x 256 pixels).
(AVI)

S3 Movie Expression of G-CaMP7 and DsRed2 imaged in the hippocampal area CA1 of TRE-G-CaMP7 transgenic mice crossed with CaMKII-tTA driver mice. Images were acquired 0 to 200 μm from the hippocampal surface at 5-μm intervals. Grayscale images of the G-CaMP7 and DsRed2 signals are shown on the left and right, respectively. Depths are indicated in the upper left corner of the movie. The size of the imaged area is 254 x 254 μm (512 x 512 pixels).
(AVI)

S4 Movie Spontaneous activity of hippocampal CA1 circuits imaged using TRE-G-CaMP7 transgenic mice crossed with CaMKII-tTA driver mice. The first 400 frames (~172 s) of the data presented in Fig 4 are shown in this movie. The playback speed is 5x faster than the speed of real activity. Time stamps are indicated in the upper left corner of the movie. The size of the imaged area is 169 x 169 μm (256 x 256 pixels).
(AVI)
