# Supplementary figures and images for: Generation and Imaging of Transgenic Mice that Express G-CaMP7 under a Tetracycline Response Element
Source: PLoS One. 2015 May 6;10(5):e0125354. doi: 10.1371/journal.pone.0125354 (PMC4422725; doi:10.1371/journal.pone.0125354)

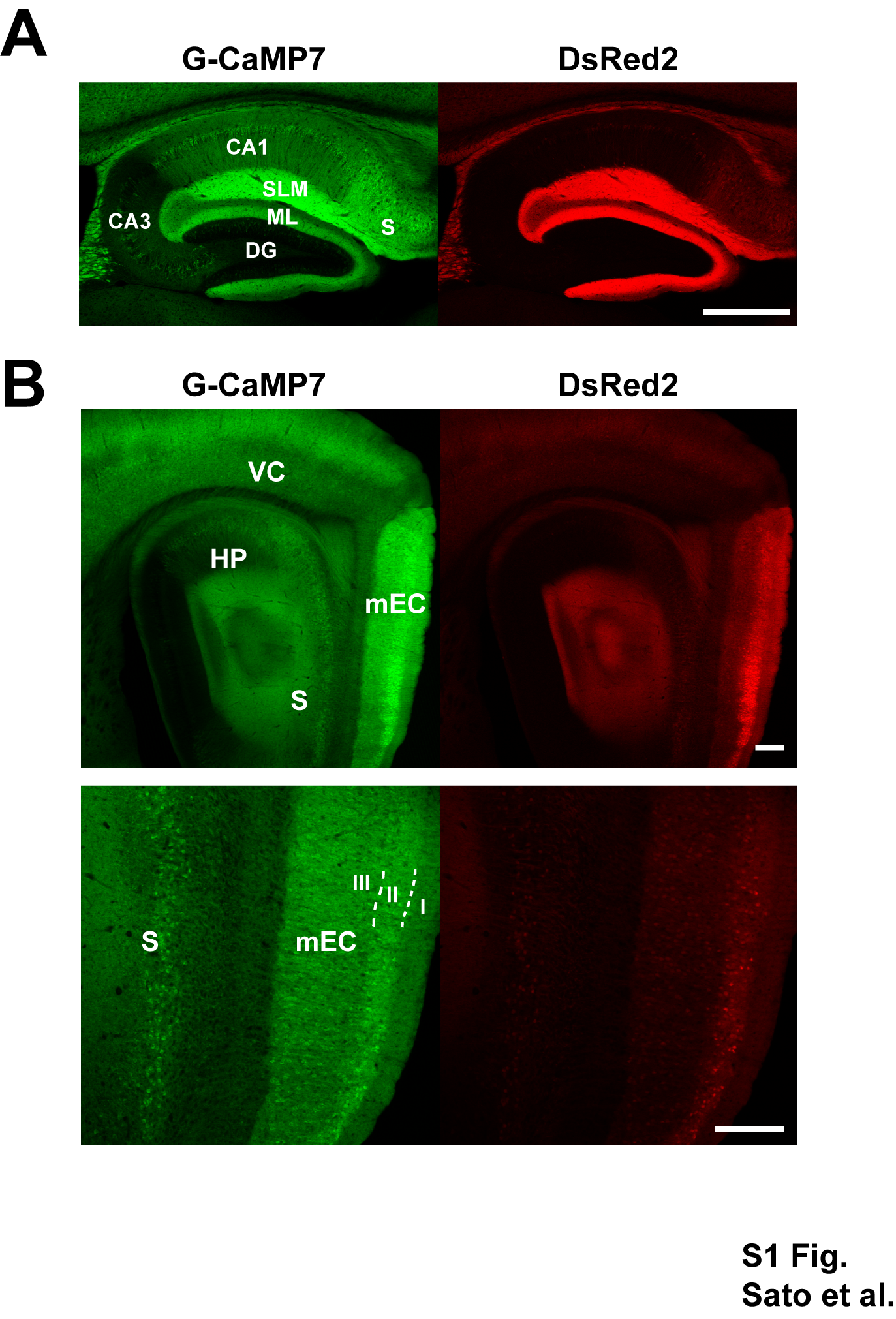

Supplement: S1 Fig — A, Expression patterns of G-CaMP7 and DsRed2 in a parasagittal section of the hippocampus. CA1, CA1 area of the hippocampus; CA3, CA3 area of the hippocampus; DG, dentate gyrus; ML, molecular layer of the dentate gyrus; SLM, stratum lacunosum-moleculare; S, subiculum. Scale bar = 500 μm. B, Expression patterns of G-CaMP7 and DsRed2 in a parasagittal section of the medial entorhinal cortex. Lower panels show higher-magnification images. HP, hippocampus; mEC, medial entorhinal cortex; I, II, and III, layers I, II, and III of the medial entorhinal cortex, respectively; S, subiculum; VC, visual cortex. Scale bar = 250 μm. (TIF) [file pone.0125354.s001.tif]

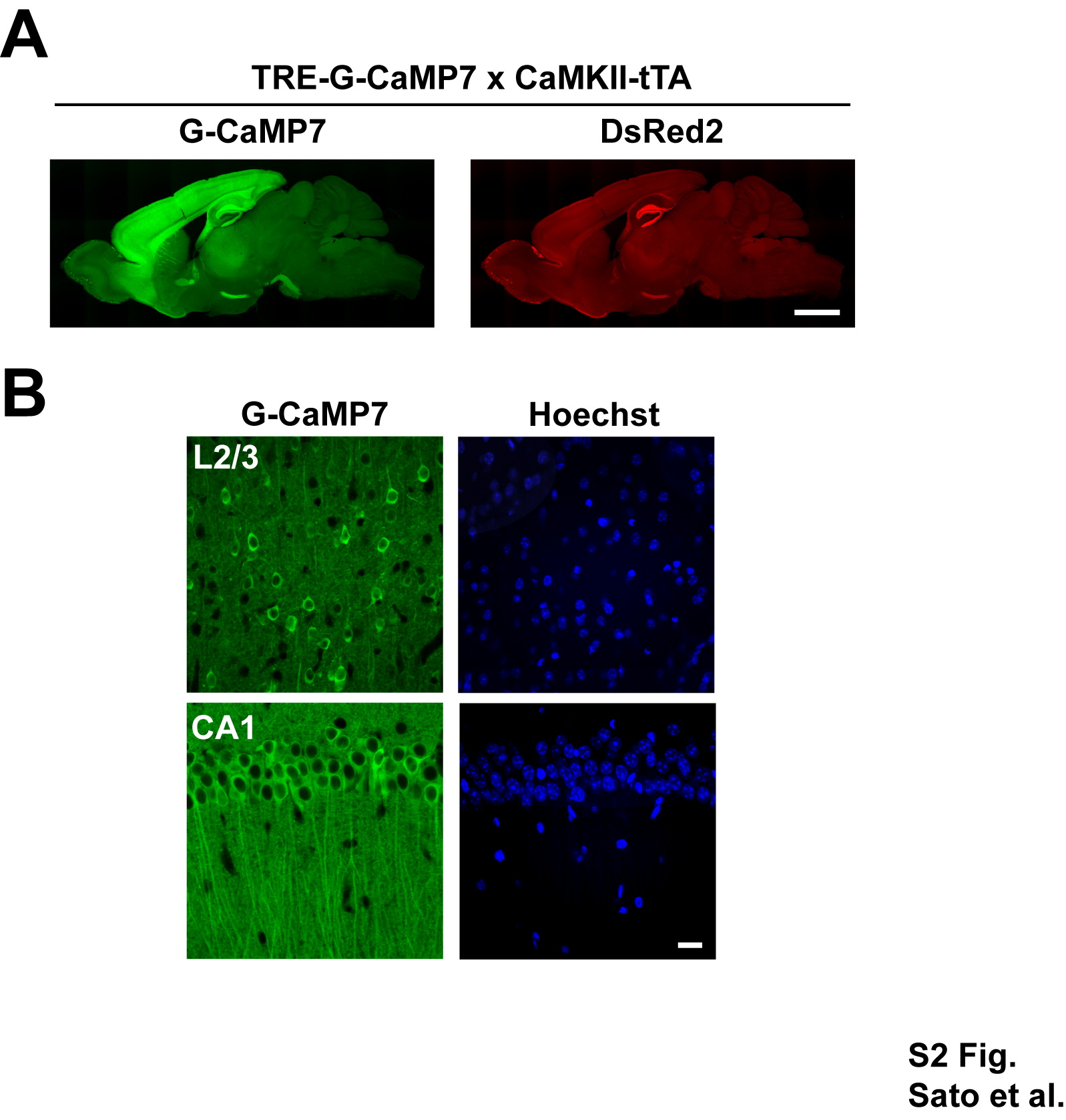

Supplement: S2 Fig — A, Expression patterns of G-CaMP7 and DsRed2 in 7-month-old mice. Scale bar = 2 mm. B, G-CaMP7 expression and nuclear staining (Hoechst) of layer 2/3 (L2/3) pyramidal neurons in the visual cortex and CA1 pyramidal neurons in the hippocampus. Scale bar = 20 μm. (TIF) [file pone.0125354.s002.tif]

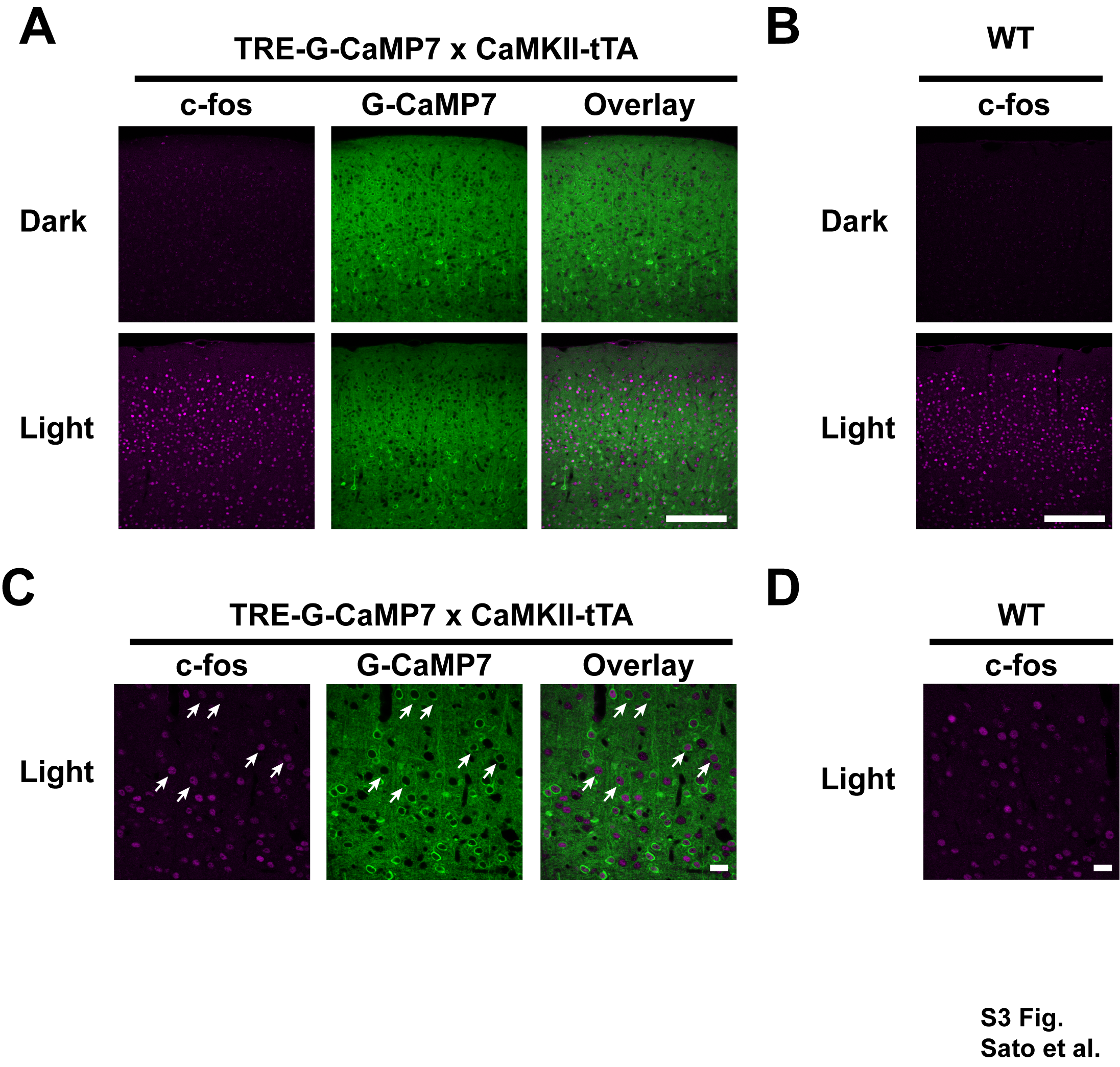

Supplement: S3 Fig — A, Light-induced c-fos expression in the visual cortex of TRE-G-CaMP7 x CaMKII-tTA mice at 8 months of age. The left, middle, and right panels show images of c-fos immunoreactivity, G-CaMP7 fluorescence and c-fos immunoreactivity overlaid with G-CaMP7 fluorescence, respectively. Top, images after 24 h of adaptation to darkness (Dark). Bottom, images after 24 h of adaptation to darkness followed by 1 h of exposure to light (Light). Scale bar = 200 μm. B, Light-induced c-fos expression in the visual cortex of wild-type mice (WT) at 8 months of age. Scale bar = 200 μm. C, Higher-magnification images of layer 2/3 visual cortical neurons expressing c-fos in response to light stimulation in TRE-G-CaMP7 x CaMKII-tTA mice at 8 months of age. Arrows indicate examples of cells that exhibited robust c-fos induction despite the presence of intracellular G-CaMP7 aggregates. Scale bar = 20 μm. D, Higher-magnification images of layer 2/3 visual cortical neurons expressing c-fos in response to light stimulation in wild-type mice at 8 months of age. Scale bar = 20 μm. (TIF) [file pone.0125354.s003.tif]

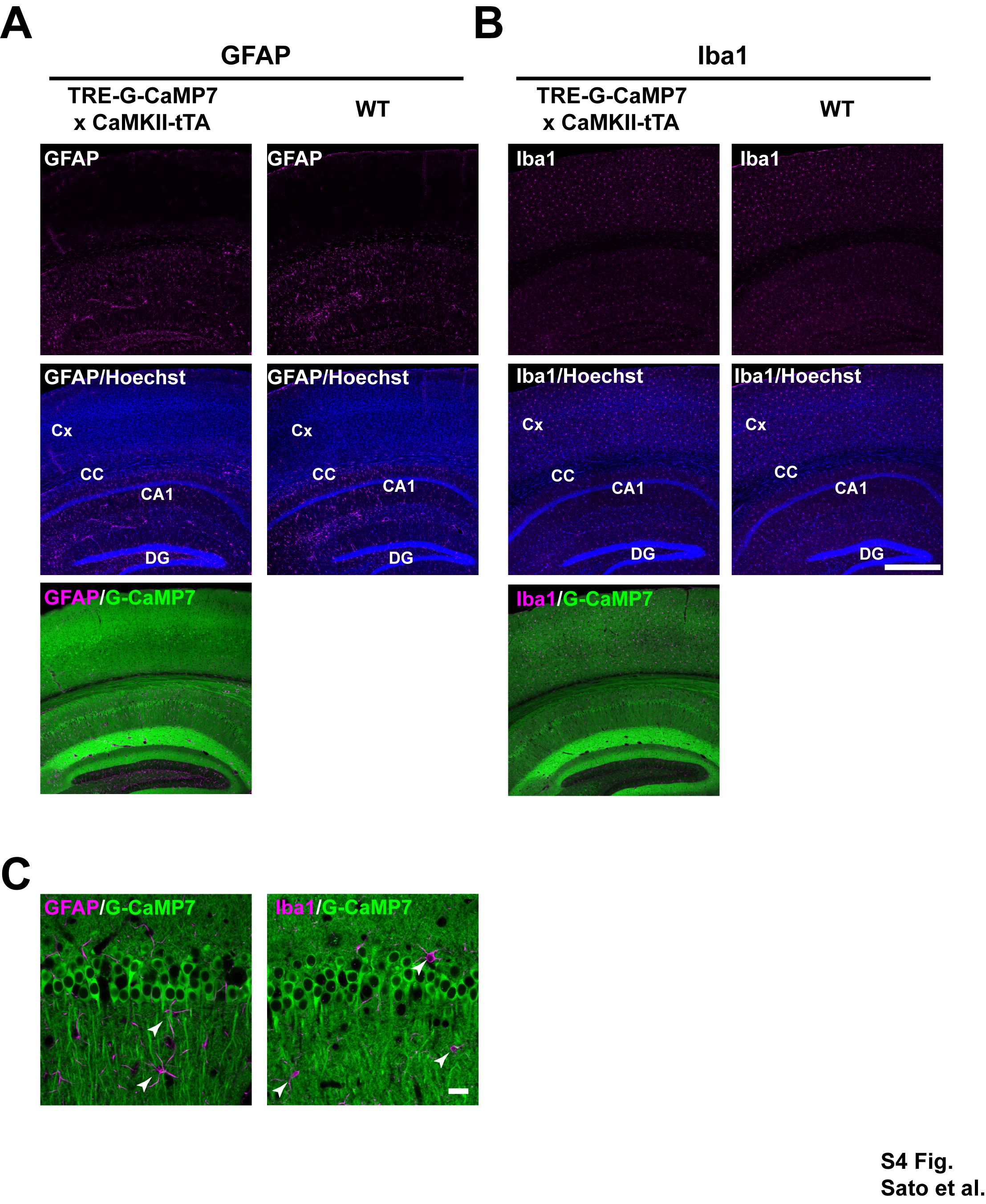

Supplement: S4 Fig — A, Top, images of GFAP immunofluoresence (magenta) in the neocortex and hippocampus of TRE-G-CaMP7 x CaMKII-tTA and wild-type (WT) mice. Middle, GFAP immunofluorescence overlaid with Hoechst nuclear counterstaining (blue). Bottom, GFAP immunofluorescence overlaid with G-CaMP7 fluorescence (green) in TRE-G-CaMP7 x CaMKII-tTA mice. B, Top, images of Iba1 immunofluoresence (magenta) in the neocortex and hippocampus of TRE-G-CaMP7 x CaMKII-tTA and wild-type mice. Middle, Iba1 immunofluorescence overlaid with Hoechst nuclear counterstaining (blue). Bottom, Iba1 immunofluorescence overlaid with G-CaMP7 fluorescence (green) in TRE-G-CaMP7 x CaMKII-tTA mice. CA1, CA1 area of the hippocampus; CC, corpus callosum; Cx, neocortex; DG, dentate gyrus. Scale bar = 500 μm. C, Higher-magnification images of GFAP-positive astrocytes (left, arrowheads) and Iba1-positive microglia (right, arrowheads) in the CA1 area of the hippocampus overlaid with G-CaMP7 fluorescence. Scale bar = 20 μm. (TIF) [file pone.0125354.s004.tif]

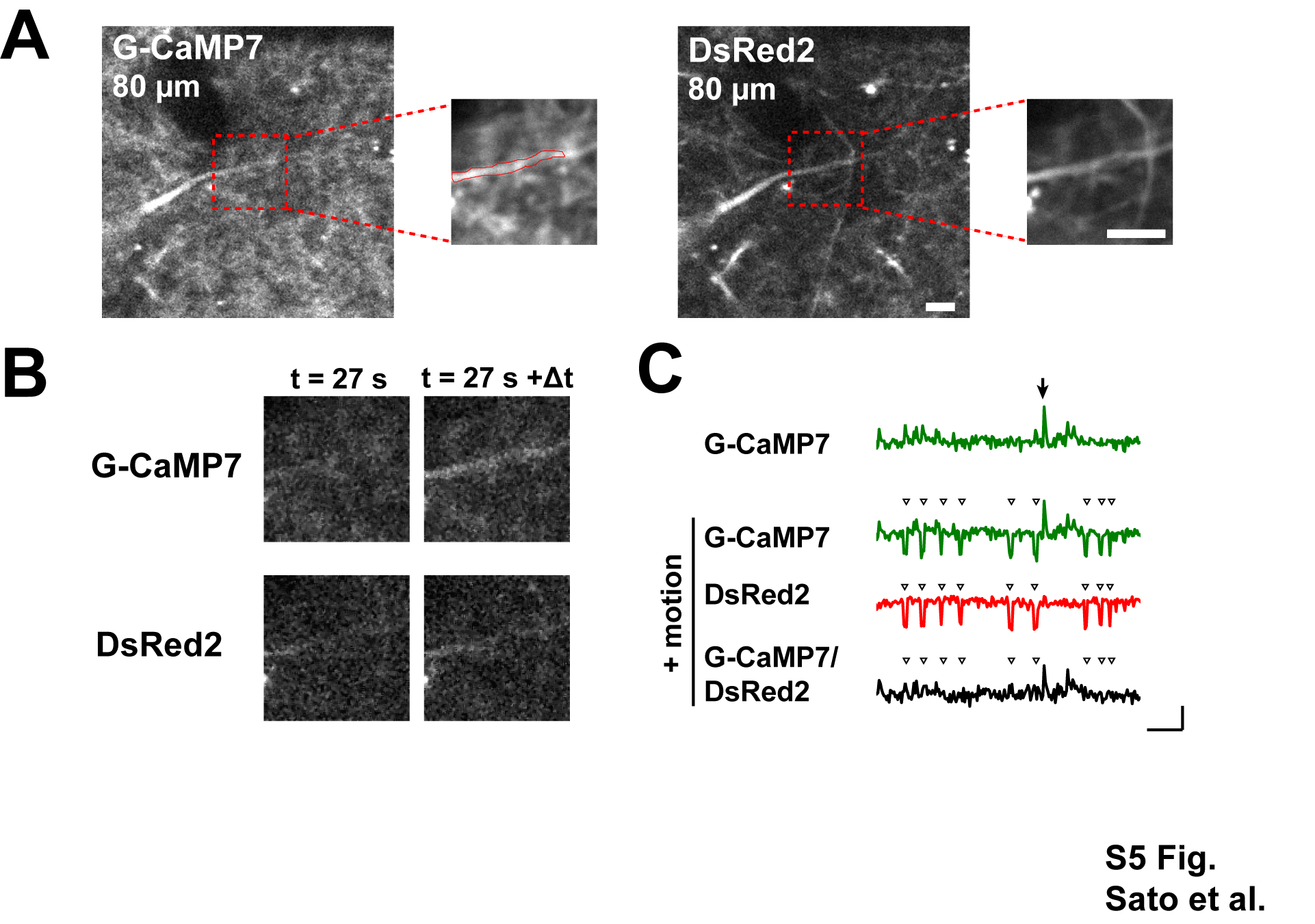

Supplement: S5 Fig — A, Basal dendrites of hippocampal CA1 pyramidal neurons labeled with G-CaMP7 and DsRed2 were imaged 80 μm from the hippocampal surface in a 6-month-old mouse. Magnified images of the areas enclosed by red dotted lines are shown to the right. The dendritic segment enclosed by the red line in the magnified G-CaMP7 image delineates the region of interest defined for the traces shown in C. Scale bar = 5 μm. B, Example time-lapse images of G-CaMP7 and DsRed2 fluorescence during spontaneous activity of a basal dendrite of a hippocampal CA1 pyramidal neuron. Δt represents the image sampling interval (0.19 s). C, Top, a trace of changes in G-CaMP7 fluorescence in the basal dendritic segment shown in A and B. The dendritic activity shown in B occurred at the time indicated by the arrow. Bottom, traces of G-CaMP7, DsRed2 and G-CaMP7/DsRed2 ratiometric signals of the same data, except for the addition of simulated motion artifacts. Artificial image displacements occurred at the random timings indicated by the inverted open triangles. Quasi-ratiometric calculations using DsRed2 signals effectively removed the baseline motion artifacts. Scale bar = 5 s (horizontal) and 20% change in the fluorescence intensity or the G-CaMP7/DsRed2 ratio (vertical). (TIF) [file pone.0125354.s005.tif]
